# A role of splenic heme biosynthesis pathway in the persistent prophylactic actions of arketamine in lipopolysaccharide-treated mice

**DOI:** 10.1038/s41398-023-02564-6

**Published:** 2023-07-25

**Authors:** Li Ma, Long Wang, Youge Qu, Xiayun Wan, Kenji Hashimoto

**Affiliations:** 1 Division of Clinical Neuroscience, Chiba University Center for Forensic Mental Health, Chiba, 260-8670 Japan; 2 Department of Anesthesiology, Renmin Hospital of Wuhan University, 430060 Wuhan, Hubei Province China

**Keywords:** Depression, Clinical pharmacology

## Abstract

Relapse is common in remitted patients with major depressive disorder (MDD). Arketamine, an (*R*)-enantiomer of ketamine, has persistent prophylactic actions in an inflammatory model of depression. However, the precise mechanisms underlying these prophylactic actions remain unknown. Given the role of the brain–spleen axis in depression, we sought to identify splenic molecular targets that play a role in the prophylactic actions of arketamine. Lipopolysaccharide (LPS) (1.0 mg/kg) was administered 6 days after a single injection of arketamine (10 mg/kg) or saline. RNA-sequencing analysis found altered expression in the heme biosynthesis II pathway. Quantitative RT-PCR revealed that pretreatment with arketamine blocked increased expression of genes involved in the heme biosynthesis II pathway in LPS-treated mice, namely, 5-aminolevulinase synthase 2 (*Alas2*), ferrochelatase (*Fech*), hydroxymethylbilane synthase (*Hmbs*). Interestingly, there were positive correlations between the expression of these genes and spleen weight or plasma levels of pro-inflammatory cytokines. We also found higher expression of ALAS2 and FECH in the spleen from MDD patients. Pretreatment with a key intermediate precursor of heme, 5-aminolaevulinic acid (300 mg/kg/day for 3 days), caused splenomegaly, higher plasma levels of pro-inflammatory cytokines, and depression-like behavior in low-dose LPS (0.1 mg/kg)-treated mice. Interestingly, pretreatment with a heme biosynthesis inhibitor, succinyl acetone (120 mg/kg/day for 3 days), had prophylactic effects in LPS (1.0 mg/kg)-treated mice. These data suggest a novel role for the heme biosynthesis II pathway in the spleen for inflammation-related depression. Therefore, the heme biosynthesis pathway could be a new target for the prevention of relapse in MDD patients.

## Introduction

Major depressive disorder (MDD), one of the most common psychiatric disorders, is a highly recurrent illness with significant public health consequences. At least 50% of MDD patients recover from their first episode of depression and have one or more additional episodes in their lifetime [1]. Considering depression is a leading cause of disability worldwide, it is of increasing importance to prevent recurrence (or relapse) in remitted patients with MDD. Although the precise mechanisms underlying relapse are incompletely understood, accumulating evidence suggests that inflammation and immunological mechanisms might play an important role [2–5].

The *N*-methyl-D-aspartate receptor (NMDAR) antagonist (*R,S*)-ketamine can produce rapid-acting and sustained antidepressant effects in treatment-resistant patients with MDD [6–11]. Preclinical data show that (*R,S*)-ketamine has long-lasting prophylactic effects in the chronic social defeat stress (CSDS) model [12] and lipopolysaccharide (LPS)-induced inflammation model [13]. (*R,S*)-ketamine is a racemic mixture that contains equal amounts of (*R*)-ketamine (or arketamine) and (*S*)-ketamine (or esketamine). Despite a lower affinity of arketamine for NMDAR than esketamine, preclinical data show that arketamine has greater potency and longer-lasting antidepressant-like actions in rodent models of depression [14–28]. Moreover, the side effects of arketamine are less than those with (*R,S*)-ketamine or esketamine [15, 19, 29–33]. Collectively, arketamine could be a potential drug for psychiatric and neurological disorders, including MDD [26–28, 34–36]. Interestingly, we previously reported that arketamine produced persistent prophylactic effects in LPS-treated mice [37, 38] and mice exposed to chronic restrained stress [39]. However, the precise mechanisms underlying these prophylactic actions of arketamine and (*R,S*)-ketamine remain unclear [40].

The spleen is an important immune organ that plays a crucial role in communication with the brain (known as the brain–spleen axis). Accumulating evidence has shown that the spleen might play a role in depression through immunological modulation, thereby implicating the brain–spleen axis in depression [23, 26, 41–45]. In addition to depression-like behavior, LPS is known to produce splenomegaly in mice, while there are positive correlations between spleen weight and blood levels of pro-inflammatory cytokines [37, 38, 45–47]. Thus, LPS-induced systemic inflammation is associated with splenomegaly in mice, resulting in depression-like behaviors. However, the role of the spleen in the persistent prophylactic effects of arketamine in LPS-treated mice remains unclear.

The purpose of this study was to investigate the role of the spleen in the prophylactic actions of arketamine in LPS-treated mice. First, we performed an RNA-sequencing analysis of the spleen of LPS-treated mice treated with either arketamine or saline. Furthermore, we measured the expression of a novel target in mouse spleen and postmortem spleen samples from patients with MDD. Second, we examined the effects of pharmacological manipulations of the novel target on splenomegaly, systemic inflammation, and depression-like behavior of LPS-treated mice.

## Materials and methods

### Animals

Male adult C57BL/6 mice (8 weeks old, body weight 20–25 g) were obtained from Japan SLC, Inc. (Hamamatsu, Shizuoka, Japan) and housed under controlled temperature and 12 h light/dark cycles (lights on between 07:00–19:00). Mice had ad libitum food and water. Mice were randomly assigned to each group. The experimental protocols were approved by the Chiba University Institutional Animal Care and Use Committee (Nos. 1-374 and 4-406).

### Compounds and treatment

Arketamine [or (*R*)-ketamine] hydrochloride was prepared by recrystallization of (*R,S*)-ketamine (Ketalar^®^, ketamine hydrochloride; Daiichi Sankyo Pharmaceutical Ltd., Tokyo, Japan) and D-(-)-tartaric acid, as reported previously [14]. A dose of 10 mg/kg arketamine (hydrochloride salt) was used as this dose showed rapid and sustained antidepressant-like effects in mouse models of depression [15, 17, 18]. LPS (L-4130, serotype 0111:B4; Sigma–Aldrich, St Louis, MO, USA) was dissolved in saline. A dose of 1.0 mg/kg of LPS was used as this dose caused splenomegaly, higher levels of pro-inflammatory cytokines, and depression-like behavior [13, 37, 38]. An intermediate precursor of heme, 5-aminolevulinic acid hydrochloride (5-ALA) (A0235; Tokyo Chemical Industry Co., Ltd., Tokyo, Japan), was dissolved in drinking water and administered orally (300 mg/kg/day) for 3 days before LPS injection [48]. The heme biosynthesis inhibitor, succinyl acetone (SA) (U0127; Tokyo Chemical Industry Co., Ltd., Tokyo, Japan) [49], was dissolved in saline and administrated intraperitoneally (i.p.) to mice for three consecutive days (120 mg/kg/day) before LPS injection [50].

### RNA-sequencing analysis

**Fig. 1:**
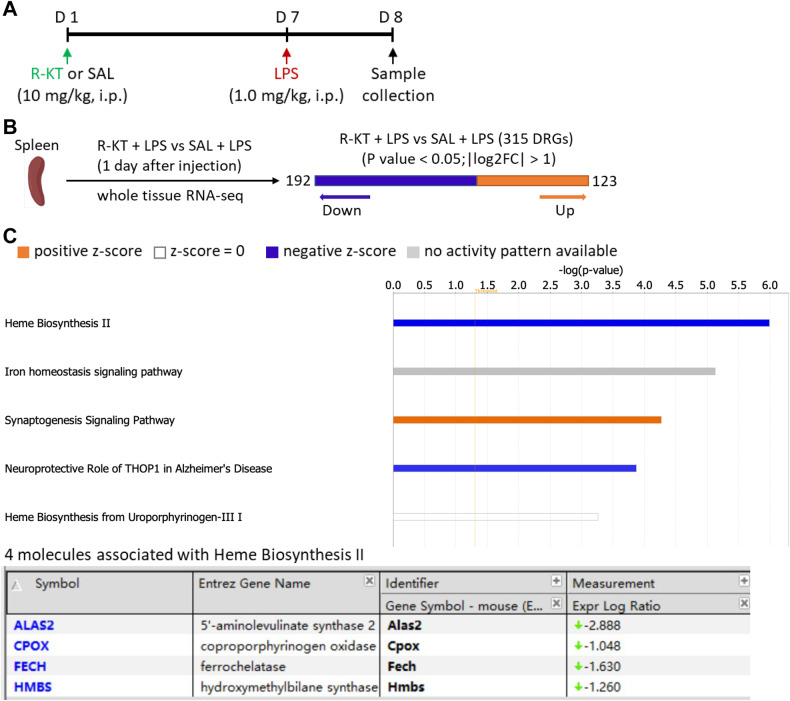
Canonical pathways of genes differentially expressed in the spleen following LPS injection. **A** Experimental protocol. Adult mice were i.p. injected with LPS (1.0 mg/kg) 6 days after i.p. administration of arketamine (10 mg/kg) or saline (10 ml/kg). **B** For RNA-sequencing, spleen samples were collected 24 h after injection of LPS. **C** Top five canonical pathways altered in the spleen were identified by Ingenuity Pathway Analysis (IPA). The heme biosynthesis II signaling pathway had the highest inhibition scores (*P* = 1.02E-06, *z*-score = −2.00). IPA *z*-score indicates whether the pathway is predicated to be inhibited (blue) or activated (red). In some cases, activation or inhibition cannot be predicted (gray).

For RNA-sequencing analysis, we used spleen samples from a previous study [37]. Briefly, arketamine (10 mg/kg) or saline (10 ml/kg) was administered i.p. to mice 6 days before a single injection of LPS (1.0 mg/kg, i.p.) (Fig. 1A). The spleen was collected 24 h after LPS injection. RNA-sequencing analysis of spleen samples was performed at Novogene (Beijing, China). The biological functions of RNA-sequencing data were analyzed using Ingenuity Pathway Analysis (IPA) [51].

### LPS-induced model and behavioral tests

The mice were randomly divided into groups. The procedure for the LPS-treated inflammation model for depression was performed as reported previously [37, 38]. Locomotion test (LMT): mice were placed in experimental cages (length, 560 × width, 560 × height, 330 mm), and locomotor activity was measured using the SCANET MV-40 (MELQUEST Co., Ltd., Toyama, Japan). Cumulative exercise was recorded for 60 min. All cages were cleaned between testing sessions. Forced swimming test (FST): mice were tested in an automated forced swim apparatus using the SCANET MV-40 (MELQUEST Co., Ltd.). The mice were placed individually in a cylinder (diameter, 23 cm; height, 21 cm) containing 15 cm of 23 ± 1 °C warm water. Immobility time was calculated by subtracting active time from total time using the apparatus analysis software. Cumulative immobility time was scored for 6 min during the test.

### Collection of blood and spleen

The mice were deeply anesthetized with inhaled isoflurane (5%) 24 h after i.p. injection of saline or LPS. Blood was collected via cardiac puncture, placed into tubes containing ethylenediaminetetraacetic acid (EDTA), and immediately centrifuged at 3000×*g* for 5 min at 4 °C to obtain plasma. Samples were then stored at −80 °C until bioanalysis, as reported previously [37]. The spleen was collected rapidly, and the spleen weight was recorded immediately. Spleen samples were stored at −80 °C until bioanalysis.

### Measurement of pro-inflammatory cytokines in the blood

Plasma levels of interleukin-6 (IL-6) and tumor necrosis factor-α (TNF-α) were determined using enzyme-linked immunosorbent assay (ELISA) kits (IL-6: 88-7064, TNF-α: 88-7324; Invitrogen, Camarillo, CA, USA) according to the manufacturer’s instructions [37].

### Quantitative real-time PCR

A quantitative RT-PCR system (Step One Plus; Thermo Fisher Scientific, Yokohama, Japan) was used. All specific mRNA transcripts were quantitatively analyzed by TaqManGene Expression assays (Thermo Fisher Scientific). Gene expression levels of 5-aminolevulinate synthase 2 (*Alas2*) (Mm00802083_m1), ferrochelatase (*Fech*) (Mm00500394_m1), hydroxymethylbilane (*Hmbs*) (Mm01143545_m1), and coproporphyrinogen oxidase (*Cpox*) (Mm00483982_m1) were measured. Total RNA was extracted using an RNase-Free DNase Set and an RNeasy Mini Kit (Qiagen, Hilden, Germany). The purity of total RNA was assessed using the BioPhotometer Plus (Eppendorf, Hamburg, Germany). cDNA libraries were obtained by reverse transcription-PCR using a High-Capacity cDNA Reverse Transcription Kit (#4368813; Thermo Fisher Scientific). All samples were analyzed twice, and arithmetic means were used for quantification. Arithmetic mean data were normalized to VIC-labeled β-actin (*Actb*) mRNA (#4352341E: pre-developed TaqMan Assay Reagents; Thermo Fisher Scientific).

### Western blotting

Human postmortem spleen samples from controls (*n* = 15) and patients with MDD (*n* = 14) were obtained from the Stanley Foundation Brain Collection (Bethesda, MD, USA). The demographic, clinical, and storage information for the cases have been previously published [42, 52, 53]. Human spleen samples were stored at −80 °C until biochemical analyses.

Tissue samples were homogenized in ice-cold Laemmli lysis buffer and then centrifuged at 3000×*g* for 10 min at 4 °C to obtain supernatants. Protein concentrations were measured using a bicinchoninic acid (BCA) protein assay kit (Bio-Rad, Hercules, CA, USA). Proteins were separated using 10% sodium dodecyl sulfate–polyacrylamide gel electrophoresis (SDS–PAGE) gels (Mini-PROTEAN^®^ TGX™ Precast Gel; Bio-Rad) and then transferred onto polyvinylidene difluoride (PVDF) membranes using a Mini Trans-Blot Cell (Bio-Rad). The membranes were blocked with 5% skimmed milk in Tris-buffered saline (TBS) with 0.1% Tween 20 (TBST) for 1 h at room temperature and then incubated with the following primary antibodies: ALAS2 (1:1000, ab184964; Abcam, Cambridge, UK), FECH (1:1000, ab137042; Abcam), HMBS (1:1000, ab129092; Abcam), and β-actin (1:10,000; Sigma–Aldrich Co., Ltd., St Louis, MO, USA) overnight at 4 °C. The membranes were washed with TBST and incubated with horseradish peroxidase (HRP)-conjugated anti-rabbit or anti-mouse antibody (1:5000) for 1 h at room temperature. After washing three times with TBST, the bands were visualized with enhanced chemiluminescence (ECL) using a Western Blot Detection system (GE Healthcare Bioscience, Chicago, IL, USA) and ChemiDoc™ Touch Imaging System (170–01,401; Bio-Rad). The images were subjected to grayscale analysis using Image Lab™ 3.0 software (Bio-Rad).

### Statistical analysis

The results are shown as mean ± standard error of the mean (SEM). Analysis was performed using PASW Statistics 20 (formerly SPSS Statistics; IBM Corp., Armonk, NY, USA). Statistical analyses were conducted by one-way analysis of variance (ANOVA), followed by post hoc Tukey test. The data using postmortem spleen samples were analyzed using Mann–Whitney *U*-test. Correlation was determined by Pearson correlation. *P* < 0.05 was considered statistically significant.

## Results

### RNA-sequencing analysis of spleen samples from mice treated with arketamine or saline

Previously, we reported that pretreatment with arketamine attenuated splenomegaly and increased plasma levels of IL-6 and TNF-α in mice after LPS administration [37]. To identify novel molecular targets in the spleen that are responsible for the prophylactic actions of arketamine (10 mg/kg, 6 days before), we examined spleen samples 24 h after a single injection of LPS (1.0 mg/kg) (Fig. 1A). We conducted RNA-sequencing analysis of spleen samples from mice treated with either arketamine or saline (Fig. 1B). The canonical pathway results identified a total of five pathways. Among these pathways, the heme biosynthesis II signaling pathway had the highest inhibition score. The four identified genes related to the heme biosynthesis II pathway from this pathway are *Alas2*, *Cpox*, *Fech*, and *Hmbs* (Fig. 1C).

**Fig. 2:**
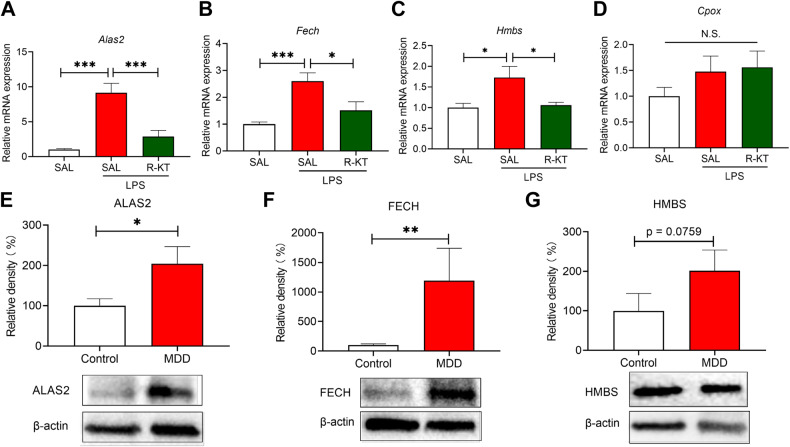
Prophylactic effects of arketamine on the expression of heme-related mRNA levels in the spleen after LPS injection. **A** *Alas2* mRNA in the spleen (one-way ANOVA: F_2,28_ = 20.61, *P* < 0.0001). **B** *Fech* mRNA in the spleen (one-way ANOVA: F_2,28_ = 10.04, *P* = 0.0005). **C** *Hmbs* mRNA in the spleen (one-way ANOVA: F_2,28_ = 5.096, *P* = 0.013). **D** *Cpox* mRNA in the spleen (one-way ANOVA: F_2,28_ = 1.207, *P* = 0.314). The data represent mean ± S.E.M. (*n* = 10 or 11). ^*^P < 0.05, ^***^P < 0.001. **E** Expression of ALAS2 in the spleen from controls and MDD patients (Mann–Whitney U-test: U = 60.50, *P* = 0.030). **F** Expression of FECH in the spleen from controls and MDD patients (Mann–Whitney U-test: U = 51, *P* = 0.030). **G** Expression of HMBS in the spleen from controls and MDD patients (Mann–Whitney U-test: U = 69.50, *P* = 0.0759). The data represent mean ± S.E.M. (control *n* = 15. MDD *n* = 14). ^*^P < 0.05, ^**^P < 0.01.

Next, we measured gene expression of *Alas2*, *Cpox*, *Fech*, and *Hmbs* in spleen samples. We found increased expression of *Alas2*, *Fech*, and *Hmbs* in the spleen from LPS-treated mice (Fig. 2A–C). Pretreatment with arketamine significantly attenuated the increased expression of these genes in LPS-treated mice (Fig. 2A–C). In contrast, there were no changes among the three groups in the expression of *Cpox* mRNA (Fig. 2D).

Western blot analysis using postmortem spleen samples showed significantly higher levels of ALAS2 and FECH in the spleen of MDD patients compared with controls (Fig. 2E, F). The expression of HMBS was also increased in the spleen of MDD patients compared with the control group but did not reach statistical significance (Fig. 2G).

### Correlations between gene expression and spleen weight or pro-inflammatory cytokines

**Fig. 3:**
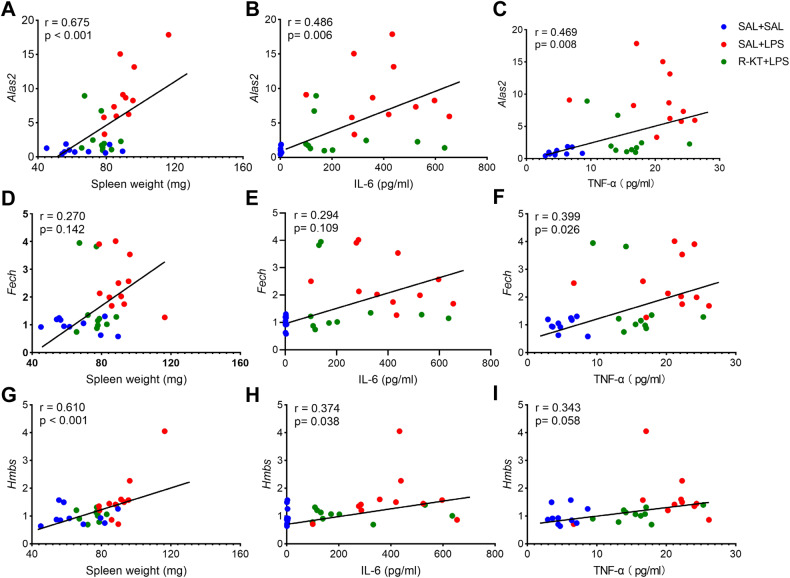
Correlation analysis of heme-related subfamily with spleen weight and pro-inflammatory cytokines. **A**–**C**: There was a positive correlation between *Alas2* mRNA and spleen weight (**A**: R = 0.675, *P* < 0.001), plasma IL-6 (**B**: R = 0.486, *P* = 0.006), or plasma TNF-α (**C**: R = 0.469, *P* = 0.008). **D**, **E** There were no correlations between *Fech* mRNA and spleen weight (**D**; R = 0.270, *P* = 0.142) or plasma IL-6 (**E**: R = 0.294, *P* = 0.109). **F** There was a correlation between *Fech* mRNA and plasma TNF-α (**F**: R = 0.399, *P* = 0.026). **G**, **H** There were positive correlations between *Hmbs* mRNA and spleen weight (**G**: R = 0.610, *P* < 0.001) or plasma IL-6 (**H**: R = 0.374, *P* = 0.038). **I** There was no correlation between *Hmbs* mRNA and plasma TNF-α (**I**: R = 0.343, *P* = 0.058). We used the data of spleen weight, plasma IL-6 and TNF-α from our previous study [37].

We found positive correlations among the three groups between *Alas2* expression in the spleen and spleen weight or pro-inflammatory cytokines (namely, IL-6 and TNF-α) (Fig. 3A–C), indicating that *Alas2* expression might be associated with systemic inflammation. Similar positive correlations were also found for *Fech* and *Hmbs* expression (Fig. 3D–I). These data suggest that gene expression of the heme biosynthesis II signaling pathway in the spleen might be associated with systemic inflammation.

### Role of splenic heme biosynthesis in LPS-induced depression-like behavior

**Fig. 4:**
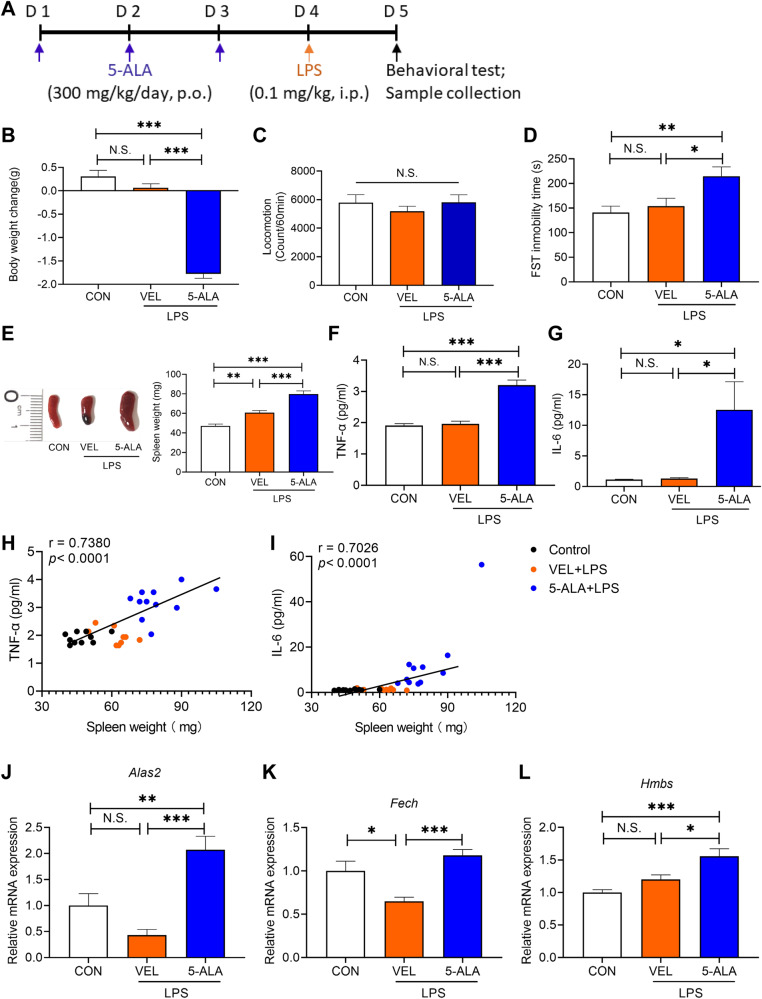
Effects of 5-ALA on depression-like phenotype, spleen weight and pro-inflammatory cytokines after injection of low dose of LPS. **A** Experimental protocol. Mice were i.p. injected with LPS (0.1 mg/kg) or saline (10 ml/kg). 5-ALA (300 mg/kg/day) or vehicle (10 ml/kg/day) was administered orally to mice for consecutive 3 days before LPS injection. Locomotion test and forced swimming test (FST) were performed 23 and 24 h after the injection of saline or LPS, respectively. Blood and spleens were collected after behavioral tests. **B** Body weight change (one-way ANOVA: F_2,28_ = 120.50, *P* < 0.0001). **C** Locomotion test (one-way ANOVA: F_2,28_ = 0.521, *P* = 0.599). **D** FST (one-way ANOVA: F_2,28_ = 6.063, *P* = 0.006). **E** Spleen weight (one-way ANOVA: F_2,28_ = 41.29, *P* < 0.0001). **F** Plasma levels of TNF-α (one-way ANOVA: F_2,28_ = 40.05, *P* < 0.0001). **G** Plasma levels of IL-6 (one-way ANOVA: F_2,28_ = 5.573, *P* = 0.009). **H** There was a positive correlation (R = 0.7380, *P* < 0.0001) between spleen weight and plasma TNF-α. **I** There was a positive correlation (R = 0.7026, *P* < 0.0001) between spleen weight and plasma IL-6. **J** *Alas2* mRNA in the spleen (one-way ANOVA: F_2,28_ = 15.77, *P* < 0.0001). **K** *Fech* mRNA in the spleen (one-way ANOVA: F_2,28_ = 11.82, *P* = 0.0002). **L** *Hmbs* mRNA in the spleen (one-way ANOVA: F_2,28_ = 10.93, *P* = 0.0003). The data represent mean ± S.E.M. (*n* = 10 or 11). ^*^P < 0.05, ^**^P < 0.01, ^***^P < 0.001. N.S., not significant.

Our current findings suggest a role of heme biosynthesis in inflammation-related depression. To investigate heme biosynthesis in the spleen, we used 5-ALA as a key metabolic intermediate of the heme biosynthesis pathway. Here, we treated mice with 5-ALA (300 mg/kg/day) for three consecutive days before LPS injection (Fig. 4A). A single low-dose injection of LPS (0.1 mg/kg) did not induce body weight loss, depression-like behavior, or an increase in pro-inflammatory cytokines in the plasma. In contrast, pretreatment with 5-ALA caused significant body weight loss in LPS-treated mice (Fig. 4B). Furthermore, pretreatment with 5-ALA significantly increased immobility time of the FST, spleen weight (Fig. 4D, E), and plasma levels of pro-inflammatory cytokines (Fig. 4F, G), but had no significant effect on locomotion (Fig. 4C). There were also positive correlations between spleen weight and plasma levels of IL-6 or TNF-α (Fig. 4H, I). Furthermore, 5-ALA upregulated gene expression levels of *Alas2*, *Hmbs*, and *Fech* in the spleen compared with the vehicle + LPS-treated group (Fig. 4J–L).

Positive correlations were found among the three groups between spleen weight and *Alas2* or *Hmbs* mRNA expression (Supplementary Fig. S1). Further, there were positive correlations among the three groups between plasma levels of IL-6 and *Alas2* or *Hmbs* mRNA expression (Supplementary Fig. S1) and plasma levels of TNF-α and *Alas2*, *Fech*, or *Hmbs* mRNA expression (Supplementary Fig. S1). These results suggest that the expression of these genes in the spleen is associated with systemic inflammation and splenomegaly in LPS-treated mice. Together, this suggests that activation of the heme biosynthesis pathway in the spleen might play a role in LPS-induced depression-like behavior through systemic inflammation.

### Inhibition of heme biosynthesis blocks LPS-induced depression-like behavior

**Fig. 5:**
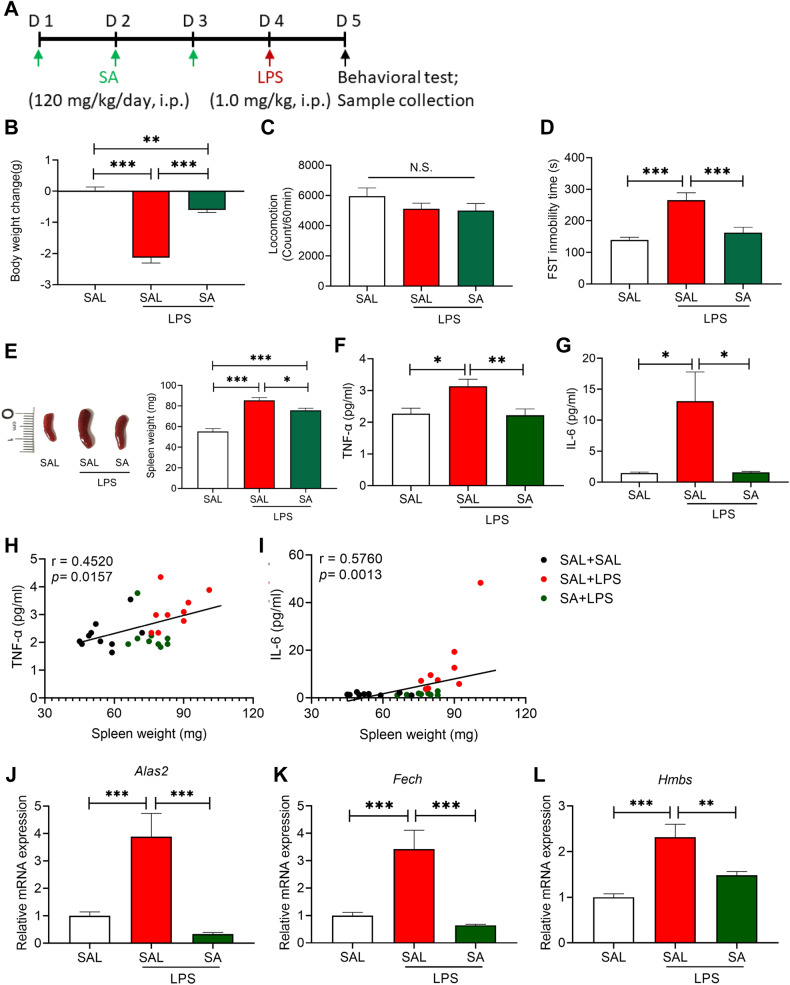
Effects of SA on depression-like phenotype, spleen weight and pro-inflammatory cytokines after LPS injection. **A** Experimental protocol. SA (120 mg/kg/day) or saline (10 ml/kg/day) was administered i.p. to mice for consecutive 3 days before injection of LPS (1.0 mg/kg) or saline (10 ml/kg). Locomotion test and forced swimming test (FST) were performed 23 and 24 h after the injection of saline or LPS, respectively. Blood and spleens were collected after behavioral tests. **B** Body weight change (one-way ANOVA: F_2,25_ = 72.50, *P* < 0.0001). **C** Locomotion test (one-way ANOVA: F_2,25_ = 1.298, *P* = 0.291). **D** FST (one-way ANOVA: F_2,25_ = 15.38, *P* < 0.0001). **E** Spleen weight (one-way ANOVA: F_2,25_ = 36.37, *P* < 0.0001). **F** Plasma levels of TNF-α (one-way ANOVA: F_2,25_ = 6.748, *P* = 0.004). **G** Plasma levels of IL-6 (one-way ANOVA: F_2,25_ = 6.456, *P* = 0.006). **H** There was a positive correlation (R = 0.452, *P* = 0.0157) between spleen weight and plasma TNF-α. **I** There was a positive correlation (R = 0.576, *P* = 0.0013) between spleen weight and plasma IL-6. **J** *Alas2* mRNA in the spleen (one-way ANOVA: F_2,25_ = 14.60, *P* < 0.0001). **K** *Fech* mRNA in the spleen (one-way ANOVA: F_2,25_ = 14.39, *P* < 0.0001). **L** *Hmbs* mRNA in the spleen (one-way ANOVA: F_2,25_ = 15.42, *P* < 0.0001). The data represent mean ± S.E.M. (*n* = 9 or 10). ^*^P < 0.05, ^**^P < 0.01, ^***^P < 0.001. N.S., not significant.

To further study the role of the heme biosynthesis pathway in LPS-induced depression-like phenotypes, we used the heme biosynthesis inhibitor, SA (Fig. 5A). Pretreatment with SA (120 mg/kg/day for 3 days) significantly attenuated LPS (1.0 mg/kg)-induced body weight loss, splenomegaly, and increased immobility time of the FST (Fig. 5B, D, E), without a significant effect on locomotion (Fig. 5C). Furthermore, pretreatment with SA significantly attenuated the increase in plasma levels of TNF-α and IL-6 in LPS-treated mice (Fig. 5F, G). There were positive correlations between spleen weight and plasma levels of TNF-α or IL-6 (Fig. 5H, I). Gene expression of *Alas2*, *Fech*, and *Hmbs* in the spleen was significantly decreased in SA-treated mice compared with saline + LPS-treated mice (Fig. 5J–L).

Positive correlations were found among the three groups between spleen weight and expression of *Alas2*, *Fech*, or *Hmbs* mRNA (Supplementary Fig. S2). There were also positive correlations between plasma levels of IL-6 and the expression of these genes (Supplementary Fig. S2). In addition, we found weak positive correlations among the three groups between plasma levels of TNF-α and *Fech* or *Hmbs* mRNA expression, although these did not reach statistical significance (Supplementary Fig. S2). These results suggest that increased expression of these genes in the spleen is associated with systemic inflammation and splenomegaly in LPS-treated mice. Further, pretreatment with the heme biosynthesis inhibitor, SA, has a prophylactic effect on LPS-induced systemic inflammation, splenomegaly, and depression-like behavior.

## Discussion

The major findings of this study are as follows: first, pathway analysis of RNA-sequencing data identified a role for the splenic heme biosynthesis II pathway in the persistent prophylactic actions of arketamine in an LPS-induced depression model. We found increased expression of three genes (*Alas2*, *Fech*, and *Hmbs*) from the heme biosynthesis II pathway in the spleen of LPS-treated mice. Interestingly, pretreatment with arketamine ameliorated increased expression of these genes. We also found positive correlations between the expression of these genes in the spleen and spleen weight or blood levels of IL-6 and TNF-α. In addition, we found increased protein expression of ALAS2 and FECH in the spleen of MDD patients compared with controls. Second, pretreatment with 5-ALA (a key intermediate of heme biosynthesis) caused body weight loss, splenomegaly, systemic inflammation, and depression-like behavior in low-dose LPS (0.1 mg/kg)-treated mice; pretreatment with vehicle did not produce these changes. Third, pretreatment with SA (an inhibitor of heme biosynthesis) significantly attenuated the body weight loss, splenomegaly, systemic inflammation, and depression-like behavior in LPS (1.0 mg/kg)-treated mice. Pharmacological data using 5-ALA and SA suggest a role for the heme biosynthesis pathway in the prophylactic effects of LPS-induced systemic inflammation and depression-like behavior. Taken together, activation of the heme biosynthesis pathway in the spleen may play a role in systemic inflammation and inflammation-induced depression-like behavior. Furthermore, arketamine may produce persistent prophylactic antidepressant-like effects by decreasing the heme biosynthesis pathway in the spleen.

It is well known that LPS produces splenomegaly in mice and that LPS-induced splenomegaly is associated with plasma levels of pro-inflammatory cytokines in these mice [37, 45–47, 54]. Thus, systemic inflammation (e.g., high levels of IL-6 and TNF-α) is associated with splenomegaly in LPS-treated mice. Despite the short half-life of arketamine in rodents [16], arketamine showed persistent (6 days) prophylactic effects in LPS-treated mice [37, 38]. Importantly, pretreatment with arketamine (6 days before) significantly attenuated splenomegaly in LPS-treated mice, despite the elimination of arketamine from the body. Thus, persistent alterations in the signaling pathway(s) induced by a single injection of arketamine might play a role in the sustained prophylactic effects of LPS-induced splenomegaly. In this study, we identified a role for the splenic heme biosynthesis II pathway for the sustained prophylactic effects of arketamine in LPS-treated mice. Interestingly, we found positive correlations between the expression of three genes (*Alas2*, *Fech*, and *Hmbs*) and spleen weight or plasma levels of pro-inflammatory cytokines. Collectively, it is likely that activation of the heme biosynthesis II pathway in the spleen may contribute to systemic inflammation, resulting in splenomegaly. The data using pharmacological agents (5-ALA and SA) strongly suggests that the heme biosynthesis pathway might play a role in systemic inflammation, resulting in depression-like behavior in mice.

In this study, we found that pretreatment (6 days before) of arketamine could ameliorate LPS-induced splenomegaly in the mice. We previously reported that splenomegaly and depression-like behaviors in CSDS-susceptible mice could be improved after a subsequent injection of arketamine (10 mg/kg) [43]. Considering the crucial role of the spleen as an immune organ, it is likely that the brain–spleen axis might play a role in the beneficial effects of arketamine on splenomegaly in mice with depression-like behaviors [23, 26, 41, 43], although further study is needed.

The heme biosynthesis pathway plays a vital role in a number of biochemical processes because heme serves as a prosthetic group of many proteins [55, 56]. 5-ALA is a naturally occurring precursor in the heme biosynthesis pathway. In this study, we found higher expression of three genes (*Alas2*, *Fech*, and *Hmbs*) of the heme biosynthesis II pathway in the spleen of LPS-treated mice and higher expression of two proteins (ALAS2 and FECH) in the spleen from MDD patients. Given the key role of ALAS2 in erythroid heme synthesis [57], higher expression of ALAS2 in the spleen might play a role in the pathogenesis of MDD through the spleen–brain axis. Interestingly, a link between iron deficiency and depression has been suggested, and supplementation of iron improved depressive symptoms in patients with MDD [58]. Therefore, future study is needed to investigate the role of the heme biosynthesis II pathway in relapse of patients with MDD.

The heme biosynthesis pathway is an essential process in cells during the energy cycle. It has been reported that 5-ALA promotes inflammatory responses through the upregulation of toll-like receptor 4 (TLR4)/nuclear factor-κB (NF-κB) and pro-inflammatory cytokines in cells treated with LPS (10 ng/mL) [59]. In this study, we found that pretreatment with 5-ALA produced systemic inflammation, splenomegaly, and depression-like behavior in low-dose LPS (0.1 mg/kg)-treated mice, and there were positive correlations between spleen weight and plasma levels of pro-inflammatory cytokines. Considering the role of 5-ALA in inflammatory response [59, 60], pretreatment with 5-ALA may enhance systemic inflammation in low-dose LPS-treated mice, resulting in splenomegaly and depression-like behavior. However, the precise mechanisms underlying arketamine-induced reduction of the activated heme biosynthesis pathway in LPS-treated mice are currently unknown. It is also unclear how arketamine can affect 5-ALA levels in the body of LPS-treated mice.

SA is formed by the oxidation of glycine and is a potent inhibitor of heme biosynthesis [49]. It has also been reported that SA suppresses inflammatory responses through upregulation of TLR4/NF-kB and pro-inflammatory cytokines in cells treated with LPS (10 ng/mL) [59]. In this study, we found that pretreatment with SA significantly attenuated body weight loss, splenomegaly, blood levels of pro-inflammatory cytokines, and increased expression of *Alas2*, *Fech*, and *Hmbs* in the spleen of LPS-treated mice. Collectively, it is likely that SA has potent anti-inflammatory actions in LPS-treated mice, consistent with a previous report [60]. Given an endogenously produced metabolite of SA in the human body, it is likely that heme biosynthesis inhibitors such as SA might be prophylactic agents in patients with MDD. Therefore, it is of interest to investigate whether supplementation of SA can prevent relapse in remitted patients with MDD.

A nationwide database study in Taiwan demonstrated that iron deficiency anemia was associated with psychiatric disorders such as depression, anxiety, sleep disorders, and psychotic disorders. Moreover, iron supplementation in individuals with iron deficiency anemia was associated with a lower risk of psychiatric disorders [61]. It is therefore important to measure blood biomarkers (e.g., iron, expression of genes or proteins) for the heme biosynthesis II pathway in MDD patients. Given the role of iron in the heme biosynthesis pathway, it is possible that iron supplementation may reduce relapse in MDD patients with iron deficiency.

Depression has a high rate of relapse, resulting in significant public health consequences [1]. Given the sustained prophylactic actions of arketamine in rodent models of depression, it is possible that arketamine might prevent this relapse in patients with MDD. Clinical studies of arketamine in treatment-resistant patients with MDD are currently underway [26, 27]. Therefore, it is of interest to examine whether arketamine can reduce relapse in remitted patients with MDD.

In conclusion, the current study suggests that the splenic heme biosynthesis II pathway might play a role in systemic inflammation, contributing to the persistent prophylactic effects of arketamine in an inflammatory model of depression. It is likely that arketamine or heme biosynthesis inhibitors could be new prophylactic agents for inflammation-related depression in humans.

## Supplementary information

Supplemental information

## Data Availability

The RNA-sequencing data have been deposited to the NCBI Sequence Read Archive and are available at the accession number PRJNA941811.
