# Supplementary material for: A role of splenic heme biosynthesis pathway in the persistent prophylactic actions of arketamine in lipopolysaccharide-treated mice
Source: Transl Psychiatry. 2023 Jul 25;13:269. doi: 10.1038/s41398-023-02564-6 (PMC10368680; doi:10.1038/s41398-023-02564-6)
Supplement: Supplementary file 1 — Supplemental information [file 41398_2023_2564_MOESM1_ESM.docx]

**Supplemental Information**

**A role of splenic heme biosynthesis pathway in the persistent prophylactic actions of arketamine in lipopolysaccharide-treated mice**

Li Ma^1*^, Long Wang^2*^, Youge Qu^1^, Xiayun Wan^1^ and Kenji Hashimoto^1^

^1^Division of Clinical Neuroscience, Chiba University Center for Forensic Mental Health, Chiba 260-8670, Japan, ^2^Department of Anesthesiology, Renmin Hospital of Wuhan University, Wuhan 430060, Hubei Province, China.

^*^Dr. Li Ma and Dr. Long Wang contributed equally.

**
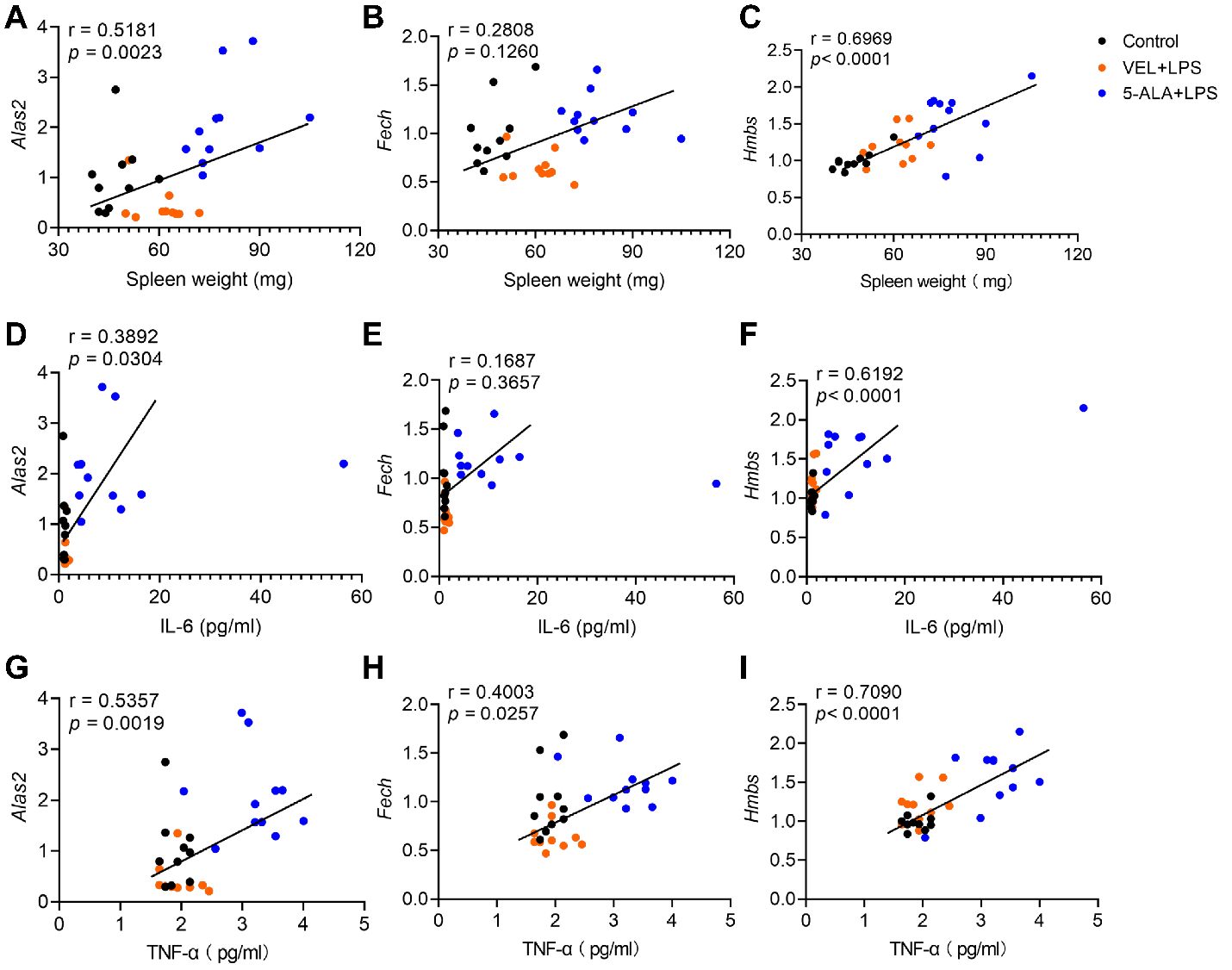
**

**Figure S1. Correlations between the expression of heme biosynthesis pathway genes in the spleen and spleen weight or plasma levels of pro-inflammatory cytokines**

(**A, C**): There were positive correlations between spleen weight and *Alas2* mRNA (**A**: R = 0.518, *P* = 0.002), or *Hmbs* mRNA (**C**: R = 0.697, *P* < 0.0001). (**B**): There was no correlation between *Fech* mRNA and spleen weight (R = 0.281, *P =* 0.126). (**D, F**): There were positive correlations between plasma IL-6 and *Alas2* mRNA (**D**: R = 0.398, *P* = 0.030), or *Hmbs* mRNA (**F**: R = 0.619, *P* < 0.0001). (**E**): There was no correlation between *Fech* mRNA and plasma IL-6 (R = 0.169, *P =* 0.366). (**G-I**): There were positive correlations between plasma TNF-α and *Alas2* mRNA (**G**: R = 0.536, *P* = 0.002), *Fech* mRNA (**H**: R = 0.400, *P* = 0.026), or *Hmbs* mRNA (**I**: R = 0.709, *P* < 0.0001).

**
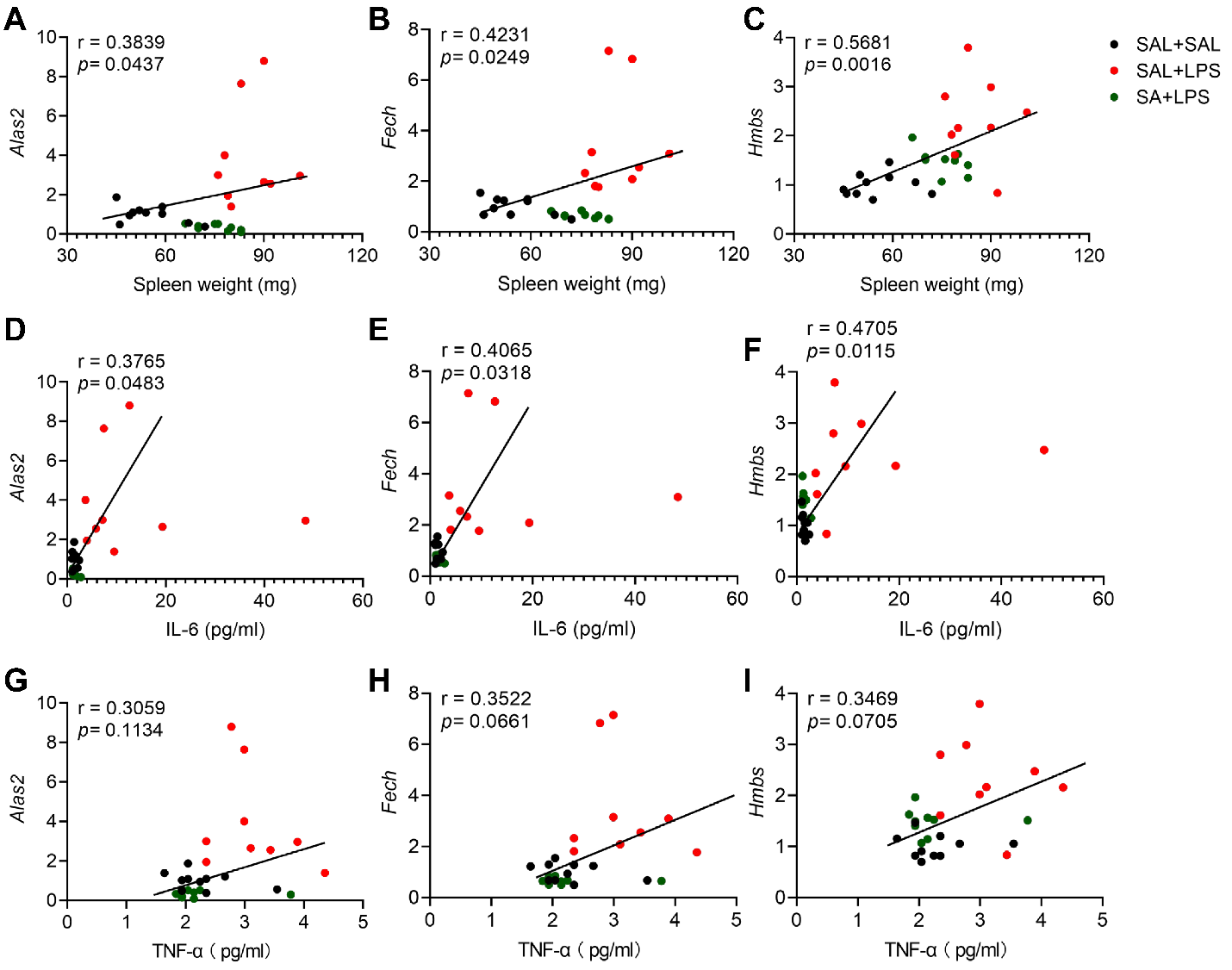
**

**Figure S2. Correlations between the expression of heme biosynthesis pathway genes and spleen weight or plasma levels of pro-inflammatory cytokines**

(**A-C**): There were positive correlations between spleen weight and *Alas2* mRNA (**A**: R = 0.384, *P* = 0.044), *Fech* mRNA (**B**: R = 0.423, *P* = 0.025), or *Hmbs* mRNA (**C**: R = 0.568, *P* = 0.002). (**D-F**): There were positive correlations between plasma IL-6 and *Alas2* mRNA (**D**: R = 0.377, *P* = 0.048), *Fech* mRNA (**E**: R = 0.407, *P* = 0.032), or *Hmbs* mRNA (**F**: R = 0.471, *P* = 0.012). (**G-I**): There were no correlations between plasma TNF-α and *Alas2* mRNA (**G**: R = 0.306, *P* = 0.113), *Fech* mRNA (**H**: R = 0.352, *P* = 0.066), or *Hmbs* mRNA (**I**: R = 0.347, *P* = 0.071).
